# Development and validation a nomogram prediction model for early diagnosis of bloodstream infections in the intensive care unit

**DOI:** 10.3389/fcimb.2024.1348896

**Published:** 2024-03-04

**Authors:** Zhili Qi, Lei Dong, Jin Lin, Meili Duan

**Affiliations:** Department of Critical Care Medicine, Beijing Friendship Hospital, Capital Medical University, Beijing, China

**Keywords:** bloodstream infections, bacteremia, intensive care unit, critically ill, early diagnosis, nomogram, prediction model

## Abstract

**Purpose:**

This study aims to develop and validate a nomogram for predicting the risk of bloodstream infections (BSI) in critically ill patients based on their admission status to the Intensive Care Unit (ICU).

**Patients and methods:**

Patients’ data were extracted from the Medical Information Mart for Intensive Care−IV (MIMIC−IV) database (training set), the Beijing Friendship Hospital (BFH) database (validation set) and the eICU Collaborative Research Database (eICU−CRD) (validation set). Univariate logistic regression analyses were used to analyze the influencing factors, and lasso regression was used to select the predictive factors. Model performance was assessed using area under receiver operating characteristic curve (AUROC) and Presented as a Nomogram. Various aspects of the established predictive nomogram were evaluated, including discrimination, calibration, and clinical utility.

**Results:**

The model dataset consisted of 14930 patients (1444 BSI patients) from the MIMIC-IV database, divided into the training and internal validation datasets in a 7:3 ratio. The eICU dataset included 2100 patients (100 with BSI) as the eICU validation dataset, and the BFH dataset included 419 patients (21 with BSI) as the BFH validation dataset. The nomogram was constructed based on Glasgow Coma Scale (GCS), sepsis related organ failure assessment (SOFA) score, temperature, heart rate, respiratory rate, white blood cell (WBC), red width of distribution (RDW), renal replacement therapy and presence of liver disease on their admission status to the ICU. The AUROCs were 0.83 (CI 95%:0.81-0.84) in the training dataset, 0.88 (CI 95%:0.88-0.96) in the BFH validation dataset, and 0.75 (95%CI 0.70-0.79) in the eICU validation dataset. The clinical effect curve and decision curve showed that most areas of the decision curve of this model were greater than 0, indicating that this model has a certain clinical effectiveness.

**Conclusion:**

The nomogram developed in this study provides a valuable tool for clinicians and nurses to assess individual risk, enabling them to identify patients at a high risk of bloodstream infections in the ICU.

## 1 Introduction

There is a significant correlation between bloodstream infections (BSI) and increased morbidity, mortality, and healthcare costs (Goto and Al-Hasan, 2013; Lydeamore et al., 2022; Verway et al., 2022).The incidence of BSIs is rising, both in the general population and among hospitalized patients (Martínez Pérez-Crespo et al., 2021). In particular, intensive care unit (ICU) patients have twice incidence of non-ICU patients (Kassaian et al., 2023). Delayed effective therapy is associated with worse outcomes (Rhodes et al., 2017; Seymour et al., 2017). Blood culture is the gold standard for diagnosing bloodstream infections, but it typically takes 1-5 days to identify microorganisms and the positivity rate of blood cultures is only 10-20% (Zhou et al., 2023; Lamy et al., 2020), This raises concerns about their cost-effectiveness. Routine blood culture waiting times limit timely escalation or de-escalation of antibiotics, contributing to the development of resistance and increased hospital costs (Cunha, 2018). Therefore, early identification of patients at high-risk for bloodstream infections may facilitate targeted blood culture collection and antibiotic administration, potentially improving patient outcomes and reducing healthcare resource consumption.

The early diagnosis of bloodstream infections focuses on two main areas: the application of molecular pathogen detection and the construction of predictive models. While a number of molecular biology techniques may facilitate early diagnosis of bloodstream infections, clinical impact has been variable, in part due to the prescriber understanding of these rapidly evolving platforms (Briggs et al., 2021). Importantly, these techniques are usually more expensive, limiting their widespread use and not completely replacing the role of blood cultures. On the other hand, predictive models often focus on predicting a bacterial with the use of various biomarkers or aim to predict the occurrence of BSI in a particular group of people (Zhao et al., 2023; Nakamura et al., 2023; Wang et al., 2021; Lee et al., 2014), and their timing is usually concentrated around the time of blood culture collection or a period preceding suspected infection (Fabre et al., 2020). Several studies have developed a machine learning (ML) algorithm to predict BSI in patients with suspected infections in the intensive care unit, but valuable predictors are mainly trends in time-series variables, which makes them geographically limited and not widely generalisable (Roimi et al., 2020; Van Steenkiste et al., 2019). Last but not least, few articles on predicting BSI have been externally validated (Jones et al., 2021; Hertz et al., 2022).

To the best of our knowledge, there are no studies predicting the risk of bloodstream infections in critically ill patients based on their admission status to the Intensive Care Unit (ICU). We aim to construct a model of bloodstream infections at the time of ICU admission and external validate the utility of the model. This model is designed to promptly identify patients at an elevated risk of developing bacteremia among all ICU admissions. It has the potential to minimize unnecessary blood culture collection in low-risk patients while guiding the appropriate use of blood cultures in the ICU.

## 2 Patients and methods

### 2.1 Patients information

This study was performed with the data from the Medical Information Mart for Intensive Care (MIMIC-IV (version 1.0) (Timmer et al., 2021; Johnson et al., 2023,) the eICU Collaborative Research Database (eICU-CRD) version 1.2 (Pollard et al., 2018). and the Beijing Friendship Hospital (BFH).MIMIC-IV database consists of comprehensive and high-quality data of patients admitted to ICUs at the Beth Israel Deaconess Medical Center between 2008 and 2019. The data in the eICU database covers patients who were admitted to a combination of many critical care units throughout the continental United States in 2014 and 2015. Both MIMIC-IV and eICU databases are public databases for critically ill patients. All data from the two database studies were extracted by the first author (certification number: 39247526), who passed the Collaborative Institutional Training Initiative examination and was granted access to the database for data extraction. The Massachusetts Institute of Technology institutional review boards approved using the database. Beijing Friendship Hospital is a tertiary hospital in Beijing, China. The validation data came from the ICU of Beijing Friendship Hospital West Side Courtyard, a comprehensive ICU with 20 beds. We selected patients who were admitted to the ICU from January 1, 2019, to June 30, 2019, and obtained the informed consent of the ethics committee of Beijing Friendship Hospital (2021-P2-053).

The MIMIC-IV and eICU data extraction codes, are available on GitHub (https://github.com). The data from BFH was collected manually. We used MIMIC-IV data for modeling. The exclusion criteria of the modeling dataset were: 1) Patients ‘age < 18 years; 2) Time to ICU admission < 48 hours; 3) Repeated admission to ICU;4) Positive blood cultures prior to admission to the ICU. As the aim of our study was to predict BSIs based on patients’ admission to the ICU, we did not exclude patients admitted to the ICU for less than 48 hours from the validation set and then performed a sensitivity analysis.

### 2.2 Definitions and diagnostic criteria

BSI is defined as the growth of a clinically significant pathogen in at least one blood culture bottle. Potential contaminants (including coagulase-negative Staphylococci, Corynebacterium species, Bacillus species, diphtheroids, Aerococcus, and Propionibacterium sp) were defined according to the Center for Disease Control and Prevention (CDC)/National Health Safety Network (NHSN) guidelines for Laboratory Confirmed Bloodstream Infections (LCBI) and were not considered BSI (Karakullukçu et al., 2017; Hall and Lyman, 2006). For patients with positive blood cultures, only the first positive result was included. Only the first blood culture result was included for patients with negative blood cultures.

### 2.3 Data collection and cleaning

We constructed the model by extracting data from the database MIMIC-IV, included 3 basic information items, 4 critical illness scores, 4 first-day vital signs, 16 laboratory test parameters, 5 baseline conditions and 15 comorbidities before ICU admission, resulting in a total of 47 characteristics. Baseline ICU assessment scales such as SOFA and Simplified Acute Physiology Score II (SAPS II) were calculated and collected on admission. Intervention events included vasopressors, mechanical ventilation, renal replacement therapy, and central venous catheterization. Laboratory information was collected on access to the ICU. We randomly divided the modeling dataset into training and internal validation datasets, which comprised 70% and 30% of the dataset, respectively.

Data cleaning: In the modeling dataset, for missing values, we first removed variables with missing values ≥20%. We then deleted all individuals with any missing values. We defined plausible ranges for vital signs. We applied the capping method to handle implausible values (**Supplementary Table 1**). In the validation dataset, we deleted all the data with missing values. In the eICU validation dataset, we also applied the capping method to handle implausible values (**Supplementary Table 2**).

The eICU website (https://eicu-crd.mit.edu/eicutables/microlab/),states that the dataset is not well populated due to limited availability of microbiology interfaces, there were only 100 blood culture-positive patients. Referring to the MIMIC-IV and BFH datasets, the proportion of BSIs was approximately 4%-5% in the included population, we matched 2000 blood culture-negative patients, resulting in the inclusion of 2100 patients as a validation set. As for admissions data of the BFH dataset, due to the manual collection by our doctors, there is no abnormal value.

### 2.4 Feature selection and modeling

Medians and interquartile ranges were used for continuous variables with a skewed distribution. In cases where two independent samples adhered to a normal distribution and variance homogeneity was assumed, we utilized the independent samples t-test for comparing differences. The chi-squared test or Fisher’s exact test was used to assess whether there was a difference between the two overall rates and constituent ratios. To establish a well-calibrated nomogram for predicting outcomes, we performed univariate regression analyses to screen for predictors, in which variables with P < 0.05 in the univariate were entered into the Lasso regression. We used variance inflation factor method to detect multicollinearity in the regression model, and we removed variables with a VIF≥4. In the Lasso regression analysis, the selected coefficient for the screening threshold is Lambda=0.025. Based on these results, we then constructed a nomogram prediction model based on independent risk factors.

The model was then validated and evaluated: Firstly, the area under the receiver operating characteristic curve (AUROC) was used to analyze the model’s accuracy. Secondly, the calibration curve is drawn through 200 repeated sampling verifications, and the correlation between the calibration curve and the standard curve is verified and evaluated. Lastly, to ascertain the model’s clinical applicability, we employed both clinical impact curve and decision curve analyses.

The analysis software for this study was R version 4.2.2 (The R Foundation for Statistical Computing, Austria, Vienna).

## 3 Results

### 3.1 Characteristics of patients

The MIMIC-IV database concludes 76540 patients, all patients included in MIMIC-IV were older than 18 years, 39,682patients were admitted to the ICU for <48h, and 125 patients had positive blood cultures before ICU admission. According to the exclusion criteria, 33993 patients remained. After removing Potential contaminants and missing values there were 1444 patients with positive blood cultures, The positive rate of blood culture was 8.72%.

To construct our models, we gathered a diverse range of data from the patient’s first day in the ICU. This included basic information items, critical illness scores, first-day vital signs, laboratory test parameters, and comorbidities and baseline conditions before ICU admission. We compared the clinical characteristics between the BSI group, blood culture negative group and without blood culture group. Patients with BSI had significantly higher rates of sepsis, days in ICU and in-hospital mortality. It can be seen that the variables such as white blood cell(WBC), ventilator use and Diabetes were statistically significant in the patients with BSI and no blood culture group but not statistically significant between the BSI and blood culture negative group. The clinical characteristics of the three groups of patients were compared in **Table 1**. The model dataset consisted of 14930 patients, including those with positive blood cultures and those without blood cultures. The training dataset consisted of 10447 patients (999 with BSI) and the internal validation dataset consisted of 4483 patients (445 with BSI). The diagram of the selection process is shown in **Supplementary Figure 1**. We divided the model dataset into train dataset and internal validation dataset, and the comparison of the clinical characteristics of these two groups of patients is shown in **Supplementary Table 3**, which shows that there is no significant difference between the train and the internal validation group of patients in terms of baseline clinical characteristics.

**Table 1:** Univariate analysis the characteristic of BSI group, negative blood culture(BC) group and no blood cultures(No-BC) group in the MIMIC-IV dataset.

Characteristic	BSI (group 1)	Negative-BC (group 2)	No-BC (group 3)	P-value (1and 2)	P-value (1and 3)
**Numbers**	1444	15112	13486		
**Age(years)**	64.0 [52.0, 73.0]	65.0 [53.0, 76.0]	67.5 [56.0, 78.0]	<0.001	<0.001
**Sex(male,%)**	866 (60.0)	8579 (56.8)	7436 (55.1)	0.02	0.001
**Weight(kg)**	79.90 [67.15, 96.00]	78.00 [65.60, 94.00]	78.00 [65.40, 92.90]	0.015	<0.001
Scoring system/index					
SAPS II(IQR)	42.00 [33.00, 53.00]	39.00[31.00, 49.00]	34.00[27.00, 42.00]	<0.001	<0.001
CCI(IQR)	6.00 [4.00, 8.00]	6.00 [4.00, 8.00]	6.00 [4.00, 8.00]	0.003	<0.001
GCS(IQR)	12.00 [7.00, 14.00]	13.00 [8.00, 14.00]	14.00[13.00, 15.00]	<0.001	<0.001
SOFA(IQR)	8.00 [5.00, 12.00]	6.00 [4.00, 9.00]	4.00 [2.00, 6.00]	<0.001	<0.001
Laboratory indicators					
Heart Rate(bpm) (IQR)	96.0 [81.0, 112.0]	91.0[78.0, 106.0]	83.0 [73.0, 96.0]	<0.001	<0.001
MAP(IQR)	78.0 [67.0, 90.0]	81.0 [70.0, 95.0]	83.0 [73.0, 95.0]	<0.001	<0.001
Respiratory Rate(IQR)	20.0 [16.0, 25.0]	20.0 [16.0, 24.0]	17.0 [15.0, 21.0]	<0.001	<0.001
Temperature(IQR)	36.83 [36.44, 37.28]	36.78 [36.44, 37.22]	36.67 [36.39, 36.94]	0.006	<0.001
Spo2(IQR)	98.0 [95.0, 100.0]	98.0[95.0, 100.00]	99.00[96.0, 100.0]	0.091	<0.001
Glucose(IQR)	131.0[104.8, 173.0]	133.0[107.0, 176.0]	130.0[106.0, 165.0]	0.059	0.447
Anion gap(IQR)	16.0 [13.0, 19.0]	15.00 [13.0, 19.0]	14.00 [12.0, 17.0]	0.001	<0.001
Bicarbonate(IQR)	21.0 [18.0, 25.0]	23.0 [20.0, 26.0]	23.0 [21.0, 26.0]	0.091	<0.001
Bun(IQR)	26.0 [16.0, 44.0]	22.0 [15.0, 38.0]	19.0 [13.0, 29.0]	<0.001	<0.001
Chloride (IQR)	102.0 [97.0, 107.0]	103.0[98.0, 107.0]	104.0[100.0, 108.0]	0.037	<0.001
Creatinine(IQR)	1.20 [0.80, 2.20]	1.10 [0.80, 1.80]	0.90 [0.70, 1.30]	<0.001	<0.001
Sodium(IQR)	137.0[134.0, 140.0]	138.0[135.0, 141.0]	139.0 [136.0, 141.0]	<0.001	<0.001
Potassium(IQR)	4.20 [3.70, 4.70]	4.20 [3.80, 4.70]	4.20 [3.80, 4.60]	0.323	0.143
Hematocrit(IQR)	31.50 [26.50, 36.62]	32.70[27.80, 38.10]	33.80 [28.50, 38.90]	<0.001	<0.001
Hemoglobin(IQR)	10.20 [8.67, 12.00]	10.60 [8.90, 12.50]	11.10 [9.30, 12.80]	<0.001	<0.001
Platelets count (IQR)	180.0[108.0, 254.0]	207.0[144.0, 284.0]	200.0[147.0, 264.0]	<0.001	<0.001
WBC(IQR)	12.15 [7.88, 18.10]	11.70 [8.10, 16.40]	10.20 [7.50, 14.00]	0.053	<0.001
RDW(IQR)	15.50 [14.00, 17.40]	14.90 [13.70, 16.70]	14.20 [13.20, 15.60]	<0.001	<0.001
PT(IQR)	15.30 [13.28, 19.60]	14.10 [12.40, 17.30]	13.40 [12.00, 15.90]	<0.001	<0.001
APTT(IQR)	32.90 [28.20, 41.52]	30.90 [27.00, 37.70]	29.90 [26.50, 35.40]	<0.001	<0.001
First Day Status					
Antibiotic Application(%)	1113 (77.1)	10830 (71.7)	7675 (56.9)	<0.001	<0.001
Renal Replacement Therapy(%)	181 (12.5)	1055 (7.0)	873 (6.5)	<0.001	<0.001
Invasive Line(%)	1118 (77.4)	10558 (69.9)	372 (2.8)	<0.001	<0.001
Vasoactive(%)	564 (39.1)	5236 (34.6)	8085 (60.0)	<0.001	<0.001
Ventilation(%)	719 (49.8)	7581 (50.2)	4343 (32.2)	0.808	<0.001
Pre-comorbidities					
Myocardial Infarct(%)	234 (16.2)	2772 (18.3)	2523 (18.7)	0.048	0.022
Congestive Heart Failure(%)	463 (32.1)	5323 (35.2)	4180 (31.0)	0.017	0.421
Peripheral Vascular Disease(%)	190 (13.2)	1839 (12.2)	1803 (13.4)	0.292	0.854
Cerebrovascular Disease(%)	235 (16.3)	2699 (17.9)	2646 (19.6)	0.141	0.002
Dementia(%)	47 (3.3)	611 (4.0)	487 (3.6)	0.163	0.536
Chronic Pulmonary Disease(%)	363 (25.1)	4237 (28.0)	3615 (26.8)	0.02	0.183
Rheumatic Disease(%)	59 (4.1)	555 (3.7)	460 (3.4)	0.471	0.209
Peptic Ulcer Disease(%)	80 (5.5)	491 (3.2)	302 (2.2)	<0.001	<0.001
Paraplegia(%)	103 (7.1)	1088 (7.2)	856 (6.3)	0.968	0.271
Renal Disease(%)	421 (29.2)	3910 (25.9)	2871 (21.3)	0.007	<0.001
Malignant Cancer(%)	262 (18.1)	2137 (14.1)	1591 (11.8)	<0.001	<0.001
Metastatic Solid Tumor(%)	97 (6.7)	1003 (6.6)	797 (5.9)	0.951	0.242
Aids(%)	15 (1.0)	146 (1.0)	73 (0.5)	0.898	0.03
Liver Disease(%)	397 (27.5)	2644 (17.5)	1022 (7.6)	<0.001	<0.001
Diabetes(%)	505 (35.0)	4830 (32.0)	4004 (29.7)	0.021	<0.001
Outcomes					
Sepsis(%)	1277 (88.4)	11714 (77.5)	5814 (43.1)	<0.001	<0.001
ICU LOS (days) (IQR)	6.34 [3.30, 13.31]	4.76 [3.01, 8.71]	3.12 [2.41, 4.42]	<0.001	<0.001
In-hospital mortality(%)	437 (30.3)	2712 (17.9)	873 (6.5)	<0.001	<0.001

SAPS II, simplified acute physiology score II; CCI, Charlson Comorbidity Index; GCS, Glasgow Coma Scale; SOFA, sepsis-related organ failure assessment; SBP, Systolic arterial pressure; DBP, diastolic blood pressure; MAP, mean arterial pressure; WBC, White blood cells count; RDW, Red blood distribution width; PT, prothrombin time; APTT, activated partial thromboplastin time; Liver Disease includes mild to severe liver disease. ICU LOS, Length of stay in the intensive care unit.

**Figure 1:**
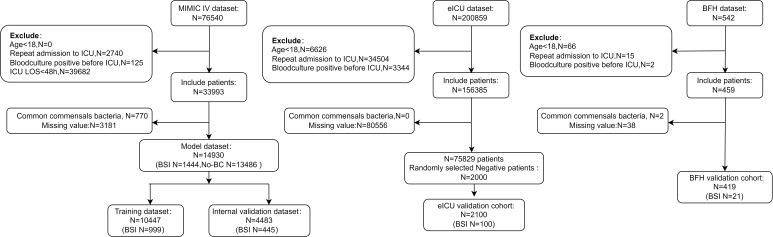
Flow diagram of blood culture data processing. ICU, Intensive Care Unit; ICU LOS, Length of stay in the ICU. Age<18, ICU LOS <48.

The eICU dataset contained 200859 patients, 75829 patients were included according to the exclusion criteria, finally, a total of 2100 patients included in the eICU validation dataset. In the BFH dataset, a total of 542 patients were admitted to the ICU during enrollment, and 419 patients were ultimately included in the validation dataset based on exclusion criteria. (**Figure 1**) In the eICU dataset 78553 patients were admitted to ICU <48 h and in the BFH dataset 243 patients were admitted to ICU <48 h. Our study aimed to predict positive blood culture results in patients on their admission status to the ICU, therefore we included all patients admitted to ICU in the validation dataset, and sensitivity analyses were performed with patients admitted to ICU <48h. **Table 2** presents a comparison of clinical characteristics among the three databases.

**Table 2:** Baseline information of the three databases.

Characteristic	Mimic iv dataset	eICU dataset	BFH dataset
**Number**	30042	2100	419
Number of positives %	1444 (4.8)	100 (4.8)	21 (5.0)
Sex,(Male %)	16881 (56.2)	1118 (53.2)	254 (60.6)
Age (years)	66.00 [54.00, 77.00]	65[53.0, 76.0]	56.0[42.0, 66.5]
First Day Status			
GCS (IQR)	14.00 [10.00, 15.00]	14.00 [10.00, 15.00]	15.00 [15.00, 15.00]
SOFA (IQR)	5.00 [3.00, 8.00]	6.00 [4.00, 8.00]	1.07 [0.00, 0.00]
Heart Rate(bpm) (IQR)	87.00 [75.00, 102.00]	88.0 [74.0, 103.0]	81.0[68.0, 99.5]
Respiratory Rate(IQR)	18.00 [15.00, 23.00]	19.0 [16.0, 23.0]	16.0 [15.0, 18.0]
Temperature(IQR)	36.72 [36.39, 37.06]	36.80 [36.40, 37.10]	36.30[36.00, 36.85]
WBC(IQR)	10.90 [7.80, 15.40]	10.50 [7.60, 14.81]	9.70 [6.50, 13.00]
RDW(IQR)	14.60 [13.40, 16.30]	14.70 [13.50, 16.30]	13.10 [12.20, 14.60]
Renal Replacement Therapy(%)	1608 (5.4)	89 (4.2)	26 (6.2)
Ventilation(%)	13198 (43.9)	338 (16.1)	330 (78.8)
Pre-comorbidities			
Myocardial Infarct(%)	5529 (18.4)	109 (5.2)	8 (1.9)
Congestive Heart Failure(%)	9966 (33.2)	178 (8.5)	44 (10.5)
Peripheral Vascular Disease(%)	3832 (12.8)	34 (1.6)	2 (0.5)
Cerebrovascular Disease(%)	5580 (18.6)	160 (7.6)	46 (11.0)
Dementia(%)	1145 (3.8)	3 (0.1)	1 (0.2)
Chronic Pulmonary Disease(%)	8215 (27.3)	195 (9.3)	2 (0.5)
Rheumatic Disease(%)	1074 (3.6)	7 (0.3)	10 (2.4)
Peptic Ulcer Disease(%)	873 (2.9)	16 (0.8)	10 (2.4)
Paraplegia(%)	2047 (6.8)	6 (0.3)	10 (2.4)
Renal Disease(%)	7202 (24.0)	214 (10.2)	18 (4.3)
Malignant Cancer(%)	3990 (13.3)	123 (5.9)	99 (23.6)
Metastatic Solid(%) Tumor(%)	1897 (6.3)	18 (0.9)	68 (16.2)
Aids(%)	234 (0.8)	5 (0.2)	0 (0.0)
Liver Disease(%)	4063 (13.5)	61 (2.9)	45 (10.7)
Diabetes(%)	9339 (31.1)	84 (4.0)	46 (11.0)
Outcomes			
Los in ICU(d)	3.83 [2.71, 6.47]	1.92 [1.04, 3.62]	1.00 [1.00, 3.00]

### 3.2 Predictors of BSI in ICU patients

**Figure 2:**
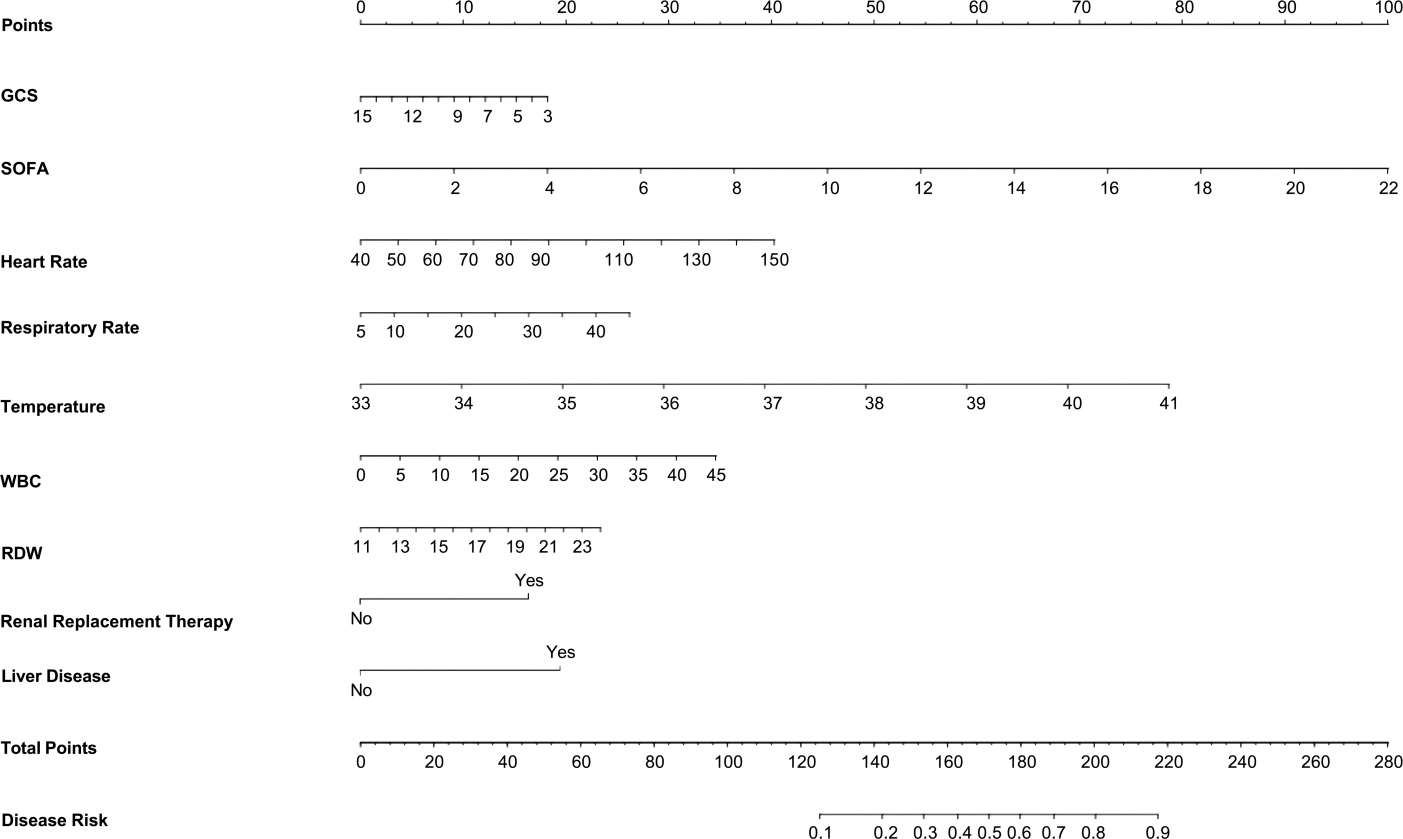
Nomogram to predict the outcomes of blood culture.

The results of the univariate analysis in the training set are shown in **Supplementary Table 4**.Variables with P < 0.05 in the univariate were entered into the Lasso regression, with a Lasso regression coefficient threshold of Lambda=0.025, we ultimately selected 9 critical variables for inclusion as shown in **Supplementary Figure 2**. These variables encompassed the GCS, SOFA, heart rate, temperature, white blood cell count, RDW, Renal Replacement Therapy, and liver disease. In this training database, a nomogram to predict the risk of bloodstream infections in critically ill patients was constructed based on their admission data to the ICU (**Figure 2**).

### 3.3 Model assessment

**Figure 3:**
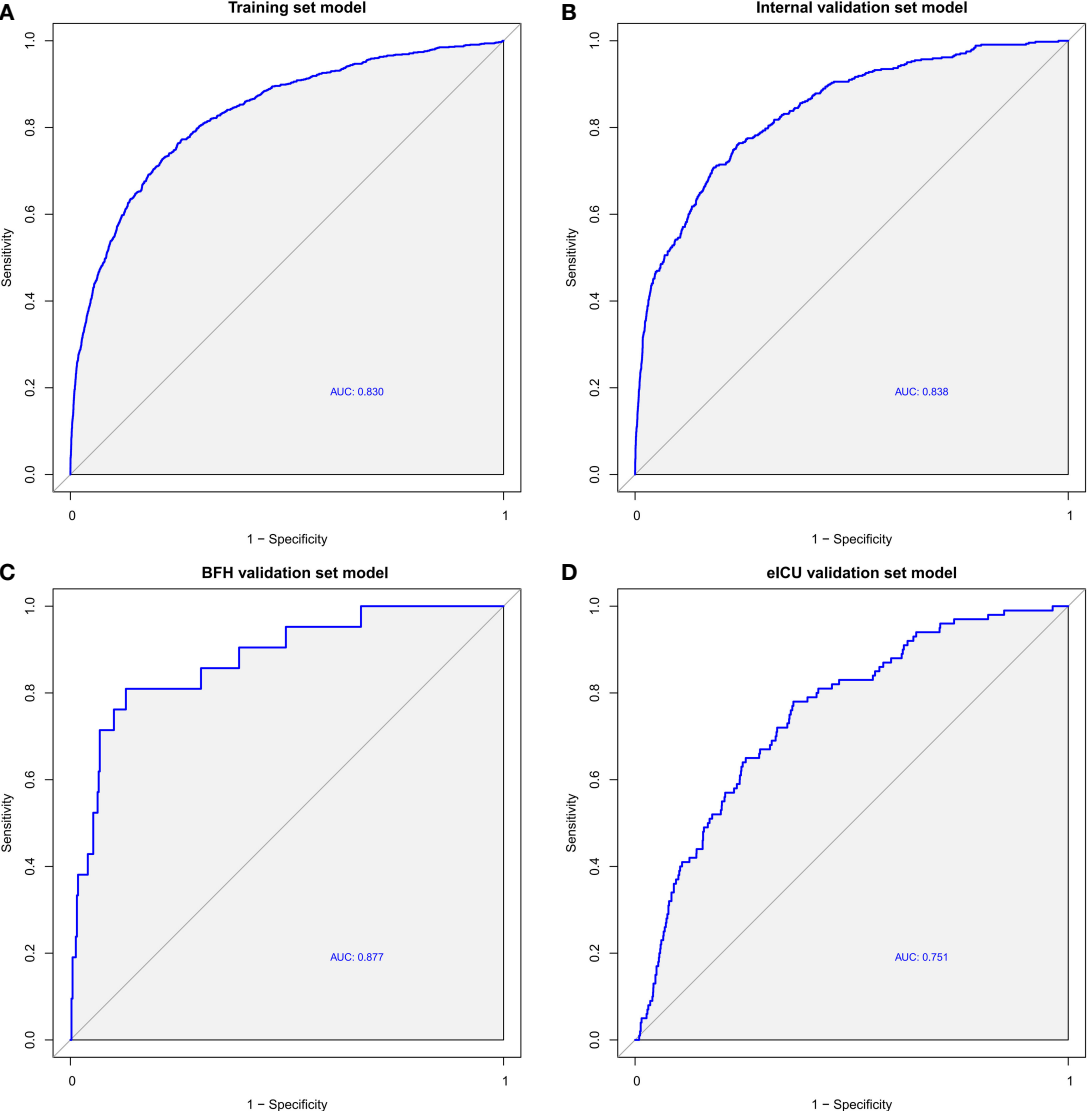
ROC curves of the Training dataset **(A)**, Internal validation dataset **(B)**, eICU validation dataset **(C)** and BFH validation dataset **(D)**.

After building the model, we evaluated its accuracy using the area under the receiver operating characteristic curve (AUROC). ROC analysis revealed an AUC (Area Under the Curve) value of 0.830 (95% CI 0.816-0.844) in the training dataset (**Figure 3A**), 0.838 (95% CI 0.818-0.858) in the internal validation dataset (**Figure 3B**), 0.878 (95% CI 0.797-0.958) in the BFH validation dataset (**Figure 3C**), and 0.751 (95% CI 0.705-0.797) in the eICU validation dataset (**Figure 3D**). These results indicate robust performance across different datasets. In addition, we excluded patients in the validation dataset who were admitted to the ICU for <48 h, and the AUROC results are shown in the **Supplementary Figure 3**, with an AUC of 0.698 (95% CI 0.653-0.756) in the eICU and an AUC of 0.838 (95% CI 0.720-0.957) in the BFH dataset.

**Figure 4:**
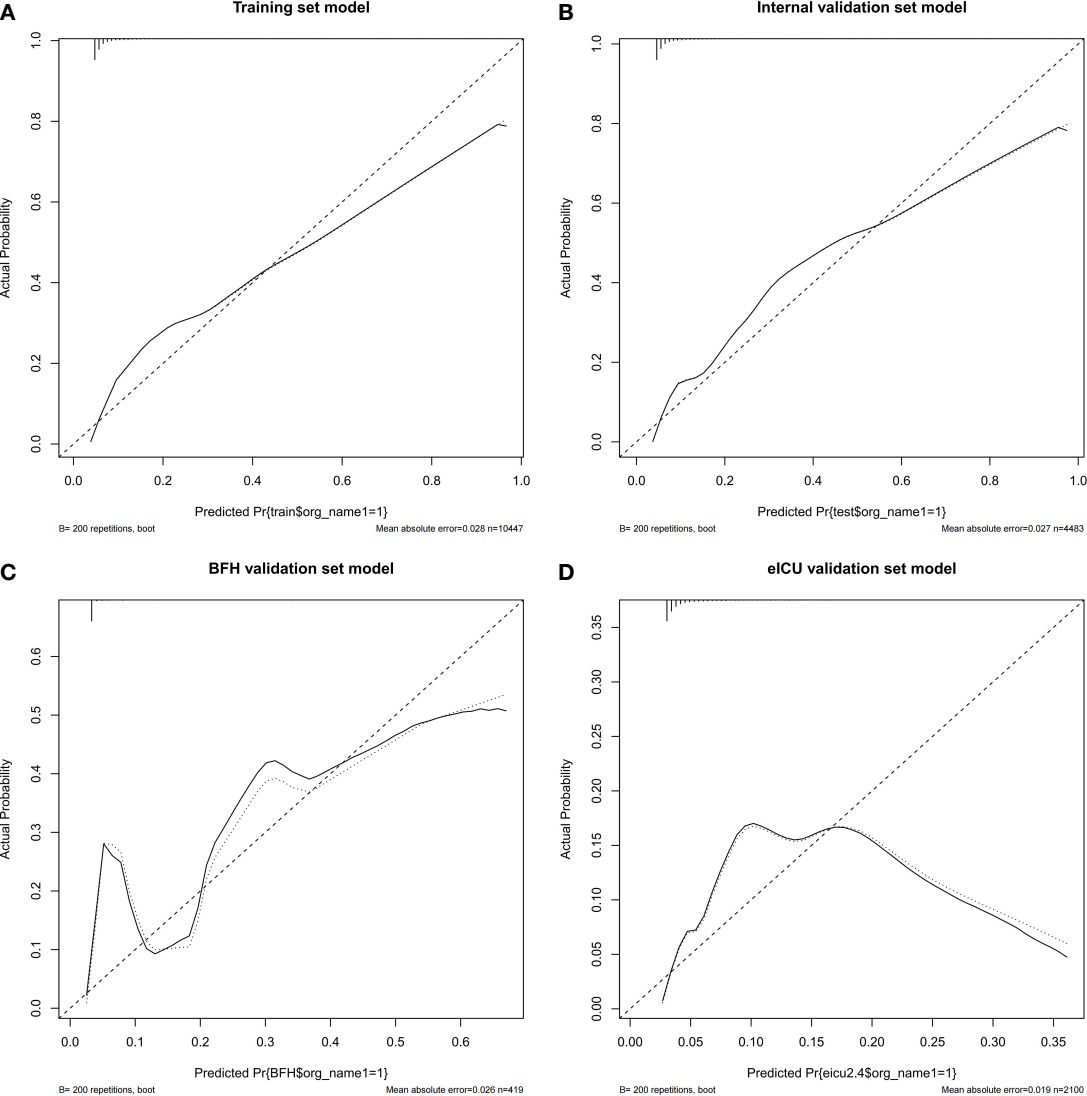
Calibration curves of the nomogram in the Training dataset **(A)**, Internal validation dataset **(B)**, eICU validation dataset **(C)** and BFH validation dataset **(D)**.

**Figure 5:**
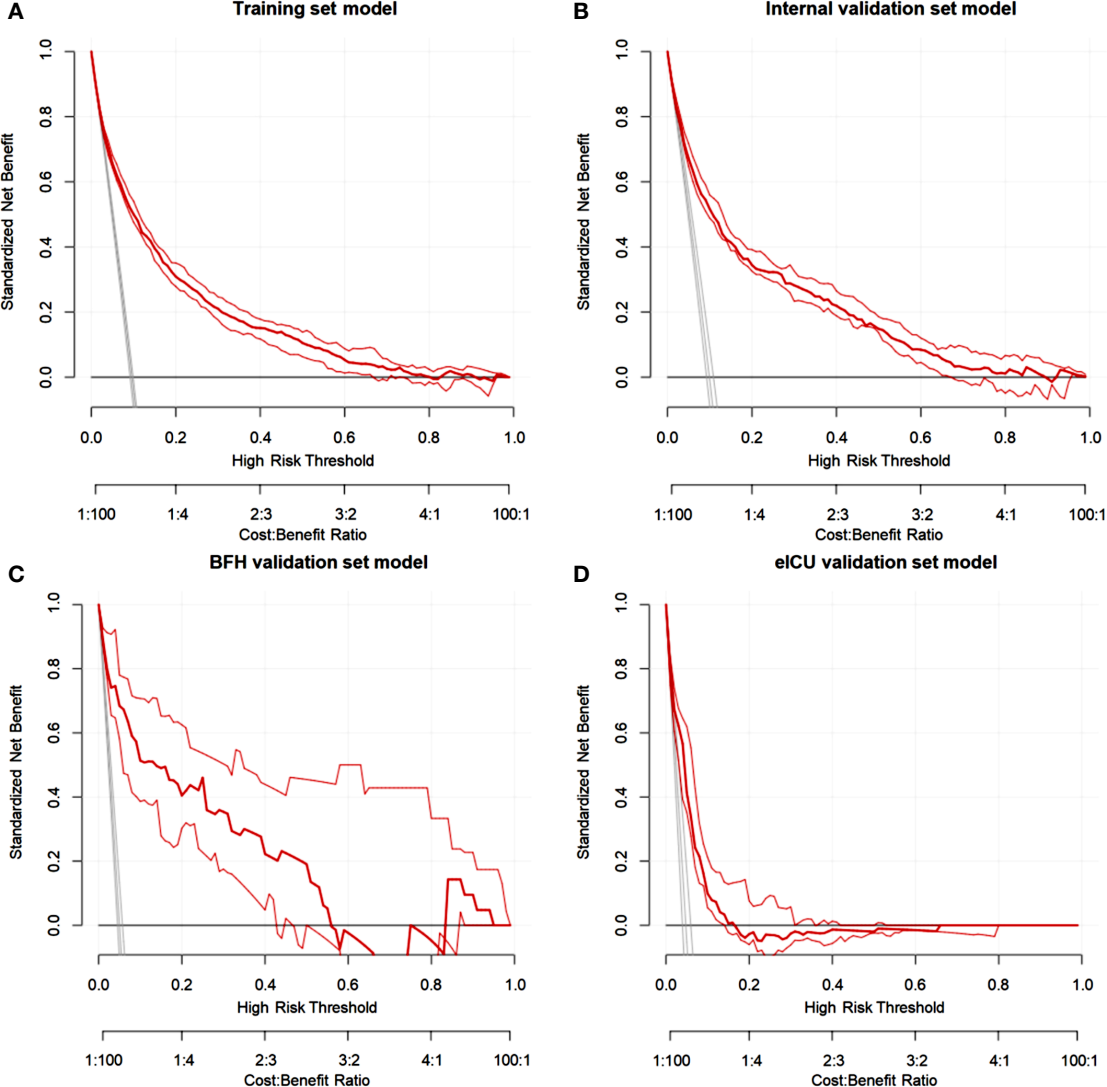
Decision curve analysis (DCA) for the nomogram in theTraining dataset **(A)**, Internal validation dataset **(B)**, eICU validation dataset **(C)** and BFH validation dataset **(D)**.

**Figure 6:**
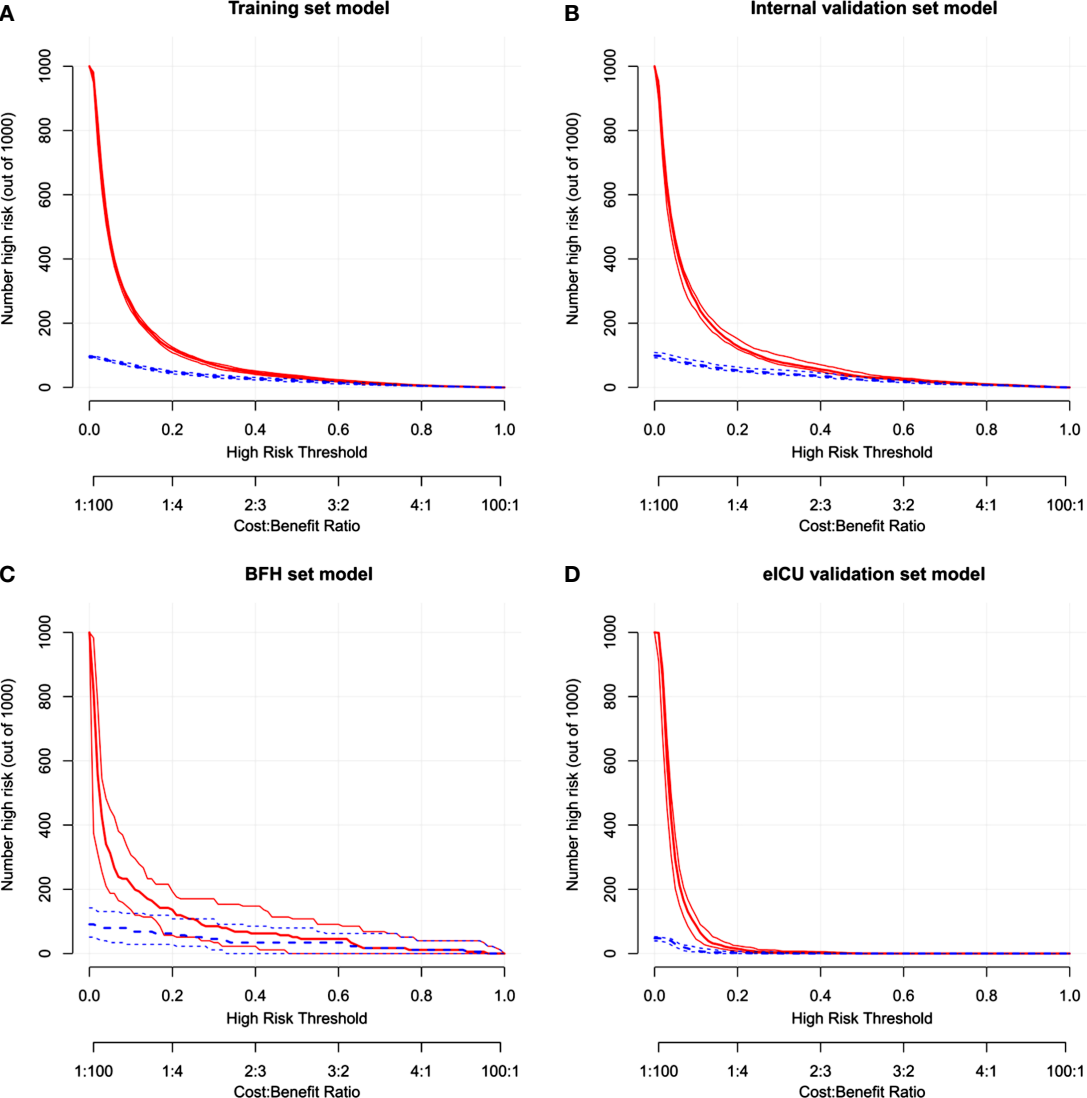
The clinical impact curve in the Training dataset **(A)**, Internal validation dataset **(B)**, eICU validation dataset **(C)** and BFH validation dataset **(D)**.

Furthermore, calibration curves were constructed to validate the effectiveness of the model further (**Figure 4**). The calibration curves showed that the prediction probabilities of the model agreed well with the BFH dataset, but did not agree well with the eICU dataset. Finally, the clinical application value of this model was further evaluated. We plotted both the decision curve (**Figure 5**) and the clinical impact curve (**Figure 6**). In the decision curves,the horizontal coordinate represents the threshold probability of predicting blood culture positive, which ranges from 0-1; the vertical coordinate represents the net benefit after benefits minus drawbacks, with a larger interval of the probability distribution of the net benefit. Three models are compared in the figure, represented by lines of three different colors. The first one is the black parallel horizontal line above the horizontal coordinate, which is the ideal model, that is, it is considered that none of the patients will develop bloodstream infections, so there is no intervention at all, and the net benefit resulting from it is 0. The second one is the gray line in the figure, which is presented in three lines, and it will show its confidence intervals. And this set of lines indicates a pessimistic attitude that blood culture positive secondary will occur in all patients. That means the net benefit through intervention when all patients are at high risk and have a poor prognosis. The third is the group of lines in red, with the thicker line in the center being the actual modeling line and its confidence interval on either side. That is, the predicted probabilities below are used to determine the circumstances under which a higher net benefit can result when going to intervention. The bottom axis represents the ratio of the payoff to the benefit curve, and it can be seen that when intervening at 40%, the payoff to benefit ratio is 2:3, and at 60%, the payoff to benefit ratio is 3:2, at which point the benefit is much smaller, suggesting that the negative impact of blood culture positive would be significant. Most of the region of the decision curve for this model is greater than 0, indicating some clinical validity. However, it should be noted that the model did not agree well with the eICU dataset.

## 4 Discussion

BSI is a significant cause of healthcare-associated infections in the ICU, with high mortality rates and increased length of stay and costs. Approximately 47.3% of ICU patients with BSIs receive inappropriate or no antibiotic treatment within the first 24 hours of onset (Tabah et al., 2012; Lee et al., 2007). Study has shown that establishing an effective predictive model can minimize unnecessary antibiotic usage in pediatric patients (Esbenshade et al., 2020). However, to the best of our knowledge, there are no studies using data collected immediately on ICU admission to predict BSI and externally validate. Our study successfully developed a predictive model for identifying the likelihood of subsequent bloodstream infections. We presented the model using a nomogram that allows assessment of the risk of bloodstream infections in ICU patients at the time of admission using commonly used available clinical data. The AUROC of the training dataset was 0.83, and the AUROCs of the external validation dataset were 0.88 and 0.75. In a few minutes or less, healthcare professionals can assess the risk of bloodstream infections in ICU patients. This provides valuable information for making timely clinical decisions based on the risk of bloodstream infections.

MIMIC-IV database is a global widely known public database in the field of critical care, many scholars have applied MIMIC-IV database for their research, which is relatively comprehensive in terms of variables, mature in terms of data extraction, and reproducible, so we applied MIMIC-IV database for the construction of the model. When Mark Verway compared patients with positive cultures with those with negative cultures, the adjusted 30-day mortality risk for positive BSIs was 1.47. Interestingly, this risk escalated to 2.62 when these patients were compared with matched datasets without blood culture testing (Verway et al., 2022). Our study also found that more variables were statistically significant in the univariate analysis in patients with BSI and those without blood cultures. So we included both patients with BSI and those without blood cultures in the modeling process. We then validated the model using data from all ICU admissions in the two external validations.

The predictive model in our study is primarily based on clinically common variables, including Glasgow Coma Scale (GCS) score, Sequential Organ Failure Assessment (SOFA) score, heart rate, respiratory rate, body temperature, white blood cell count, red cell distribution width (RDW), whether renal replacement therapy was used on day 1 and presence of liver disease. Our study includes a comprehensive array of data, including critical illness scoring, vital signs, laboratory indicators and baseline conditions. The variables we included are consistent with other studies. Temperature, white blood cells, and SOFA scores are risk factors often incorporated into predictive models of bloodstream infections (Van Steenkiste et al., 2019; Hertz et al., 2022; Schwenzer et al., 1994; Pereira et al., 2012), some articles have incorporated heart rate, respiratory rate, and the presence of renal replacement therapy (Lorente et al., 2022; Montrucchio et al., 2022; Previsdomini et al., 2012).RDW is known to be associated with mortality, an elevated RDW at admission is linked to adverse outcomes in the short and long term for both adult and neonatal patients (Dankl et al., 2022). However, RDW has limited diagnostic value for sepsis (Hu et al., 2020). Nathan Jones and Frederik Boetius Hertz analyzed the value of some biomarkers in assessing the risk of bloodstream infections, the AUC reporting on the discriminatory power between 0.5017-0.8243, but they were not conduct externally validated (Jones et al., 2021; Hertz et al., 2022). The variables in our predictive model included criticality scores, baseline conditions and laboratory indicators, which may explain the better results of our model, in addition, we validated our predictive model with an external dataset.

In our study, AUC did not declined in the BFH dataset, but declined more significantly in the eICU dataset, and we considered several reasons for this: Firstly, The eICU dataset is not well populated due to limited availability of microbiology interfaces (Pollard et al., 2018). The eICU database contains more than 200,000 patients but only 100 blood culture positive patients were ultimately included in our study, so we randomly selected 2000 blood culture negative patients to collectively form the validation set, but this may have the problem of data bias. Secondly, comparison of the variables in the three datasets revealed statistically significant differences in baseline characters, suggesting that there may be differences in the type and severity of patients admitted, which may account for the decline in AUC. The AUC of our model is above 70% in both external validation sets, In our opinion, the presence of differences in the baseline data is more conducive to expand the application of the predictive model. In the BFH data, a large proportion of patients with cerebrovascular disease and tumors can be seen, which may indicate that the model may be more valuable for extension to patients with tumors and cerebrovascular disease. Thirdly, the small number of positive results in the eICU and BFH databases may also lead to bias in the performance of the models, which might be reduced by expanding the sample size.

Our study has several limitations. First, there is insufficient baseline data for the three centers, which may not be conducive to exploring why the efficiency of the model decreases, but this does not affect the verification and use of the model. Second, we did not collect data on the source of the pathogens or variables such as procalcitonin (PCT) and albumin due to the high missing values in the MIMIC-IV database and the non-routine measurement in some centers. The inclusion of these variables could potentially improve the efficiency of the predictive model. Third, the model has not been prospectively validated. We plan to conduct a multicenter, prospective validation study of this bloodstream infection prediction model.

## 5 Conclusion

In our study, we developed a predictive model for bloodstream infections in critically ill patients on the first day of ICU admission. We employed a nomogram as a visual tool to facilitate an intuitive and practical assessment of bloodstream infection risk. This nomogram utilizes available clinical data from the patient’s first day in the ICU. Such a tool may help determine whether to initiate or modify antibiotic therapy and reduce healthcare resources. Further research should focus on improving the classification performance and conducting a larger external validation to assess the clinical impact of the model.

## Data availability statement

The original contributions presented in the study are included in the article/**Supplementary Material**. Further inquiries can be directed to the corresponding author.

## Ethics statement

The studies involving humans were approved by Bioethics Committee of Beijing Friendship Hospital, Capital Medical University. The studies were conducted in accordance with the local legislation and institutional requirements. Written informed consent for participation was not required from the participants or the participants’ legal guardians/next of kin in accordance with the national legislation and institutional requirements.

## Author contributions

ZQ: Data curation, Methodology, Software, Validation, Writing – original draft. LD: Data curation, Validation, Writing – review & editing. JL: Conceptualization, Writing – review & editing. MD: Conceptualization, Project administration, Supervision, Writing – review & editing.
