# Supplementary material for: Development and validation a nomogram prediction model for early diagnosis of bloodstream infections in the intensive care unit
Source: Front Cell Infect Microbiol. 2024 Mar 4;14:1348896. doi: 10.3389/fcimb.2024.1348896 (PMC10946253; doi:10.3389/fcimb.2024.1348896)

**Development and Validation a Nomogram Prediction Model for Early Diagnosis of Bloodstream Infections in the Intensive Care Unit**

Electronic supplementary material, Infection and Drug Resistance

**Authors:**

Department of Critical Care Medicine, Beijing Friendship Hospital, Capital Medical University, Beijing, China

**Corresponding author**:

Meili Duan, Department of Critical Care Medicine, Beijing Friendship Hospital, Capital Medical University, Beijing, China

E-mail: dmeili@ccmu.edu.cn, Cell-phone number: 86-010-63138011

Address: No. 95 Yong’an Road, Xicheng District, Beijing, 100050

Supplementary Table 1. Data cleaning of the MIMIC-IV cohort

| **Variables name** | **Value before treatment** | **Value after treatment** |
| --- | --- | --- |
| Weight（kg） | 1.00-730.00 | 38.4-177.16 |
| Heart Rate (bpm) | 26-244 | 46-152 |
| Systolic arterial pressure (mmHg) | 1-314 | 68-200 |
| Diastolic arterial pressure (mmHg) | 1-203 | 29-132 |
| Mean arterial pressure (mmHg) | 1-295 | 41-147 |
| Respiratory Rate (per minute) | 1-67 | 8-42 |
| Temperature (Celsius) | 26.3-41.20 | 33.34-41.20 |
| SPO2 | 4-100 | 78-100 |
| Glucose | 2-99999.9 | 52.11-517 |
| Anion gap | -8-56 | 7-37 |
| Bicarbonate | 2-50 | 7-40 |
| Bun | 1-260 | 4-134 |
| Calcium | 1.8-27.5 | 5.7-11.2 |
| Chloride | 46-150 | 79-150 |
| Creatinine | 0.1-43 | 0.3-10.9 |
| Sodium | 74-177 | 116-157 |
| Potassium | 1.5-10 | 2.6-8.0 |
| Hematocrit | 6.4-69.7 | 16.2-52.5 |
| Hemoglobin | 1.7-22.5 | 5.2-17.2 |
| Platelet count | 5-2947 | 19-703.6 |
| WBC | 0.1-402.50 | 0.9-46.48 |
| RDW | 10.7-33.5 | 11.8-25 |
| PT | 8-154.6 | 9.8-78.5 |
| APTT | 16.6-150 | 19.7-150 |

Note: We applied the cap blocking method to handle outliers, excluding outliers on both sides at 5%. We considered a maximum temperature of 41.2°C and SPO_2_ of 100% as reasonable. Abbreviations: RDW: red blood cell distribution width. WBC: white blood cell count. PT：prothrombin time. APTT: activated partial thromboplastin time.

Supplementary Table 2. Data cleaning of the eICU cohort

| **Variables name** | **Value before treatment** | **Value after treatment** |
| --- | --- | --- |
| Heart Rate (bpm) | 0-224 | 42-153 |
| Respiratory Rate (per minute) | 0-176 | 6-43 |
| Temperature (Celsius) | -14.4-530.5 | 33.4-39.5 |
| WBC | 0-1147193.0 | 0.9-55 |
| RDW | 0-60.9 | 11.9-25.6 |

Note: We applied the cap blocking method to handle outliers, excluding outliers on both sides at 5%. We considered a maximum SPO_2_ of 100% as reasonable. Abbreviations: RDW: red blood cell distribution width. WBC: white blood cell count.

**Supplementary Figure 1. Flow diagram of modeling cohort processing**

Note: We exclude patients aged <18 years old, admitted to the ICU <48 h,repeat admission to ICU and bloodculture positive before ICU,then exclued potential contaminants and missing values.The remaining 30042 episodes were divided into negative or positive episodes and episodes without blood culture. Patients with positive blood cultures and patients without retained blood cultures comprised the modeling cohort, which was divided into a training dataset and a validation dataset for modeling and validation.


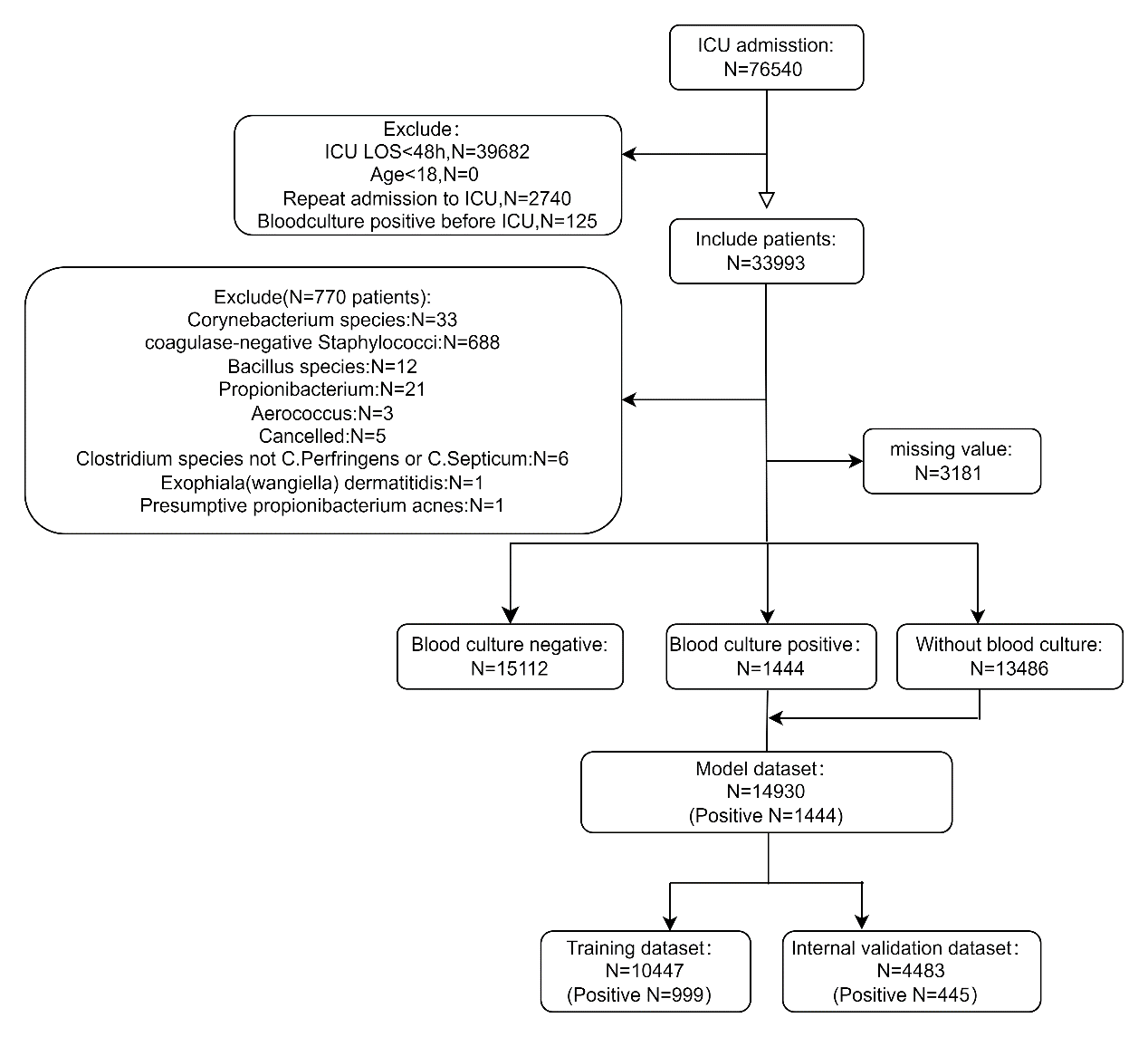


**Supplementary Table 3. Univariate Analysis of the characteristic of Training dataset and Internal validation dataset**

| **Characteristic** | **Training set** | **Internal validation set** | **Univariate Analysis**  **P value** |
| --- | --- | --- | --- |
| **Number** | 10447 | 4483 |  |
| **Number of positives %** | 999 ( 9.6) | 445 ( 9.9) | 0.51 |
| **Sex,(Male %)** | 5781 (55.3) | 2521 (56.2) | 0.32 |
| **Age (years)** | 67.00 [56.00, 77.00] | 67.00 [56.00, 78.00] | 0.265 |
| **Weight,(IQR)** | 78.30 [65.65, 93.20] | 78.00 [65.50, 92.55] | 0.285 |
| **Scoring system/index** |  |  |  |
| SAPSII (IQR) | 35.00 [27.00, 43.00] | 35.00 [27.00, 43.00] | 0.131 |
| CCI (IQR) | 6.00 [4.00, 8.00] | 6.00 [4.00, 8.00] | 0.291 |
| GCS (IQR) | 14.00 [12.00, 15.00] | 14.00 [12.00, 15.00] | 0.25 |
| SOFA (IQR) | 4.00 [2.00, 7.00] | 4.00 [2.00, 7.00] | 0.148 |
| **Laboratory indicators** |  |  |  |
| Heart Rate(bpm) (IQR) | 84.00 [74.00, 97.50] | 84.00 [73.00, 97.00] | 0.812 |
| MAP (IQR) | 83.00 [72.00, 95.00] | 83.00 [72.00, 95.00] | 0.897 |
| Respiratory Rate(IQR) | 18.00 [15.00, 22.00] | 17.00 [15.00, 22.00] | 0.147 |
| Temperature(IQR) | 36.67 [36.39, 37.00] | 36.67 [36.39, 37.00] | 0.992 |
| Spo2(IQR) | 98.00 [96.00, 100.00] | 99.00 [96.00, 100.00] | 0.317 |
| Glucose(IQR) | 130.0[106.0, 166.0] | 130.00 [106.00, 165.00] | 0.329 |
| Anion gap(IQR) | 14.00 [12.00, 17.00] | 14.00 [12.00, 17.00] | 0.255 |
| Bicarbonate(IQR) | 23.00 [21.00, 26.00] | 23.00 [21.00, 26.00] | 0.224 |
| Bun(IQR) | 19.00 [14.00, 30.00] | 19.00 [14.00, 30.00] | 0.181 |
| Chloride (IQR) | 104.00 [99.00, 107.00] | 104.00 [99.50, 108.00] | 0.348 |
| Creatinine(IQR) | 1.00 [0.70, 1.40] | 1.00 [0.70, 1.40] | 0.076 |
| Sodium(IQR) | 139.00 [136.00, 141.00] | 139.00 [136.00, 141.00] | 0.744 |
| Potassium(IQR) | 4.20 [3.80, 4.70] | 4.20 [3.80, 4.60] | 0.424 |
| Hematocrit(IQR) | 33.60 [28.40, 38.70] | 33.50 [28.15, 38.80] | 0.419 |
| Hemoglobin(IQR) | 11.00 [9.20, 12.80] | 10.90 [9.20, 12.80] | 0.348 |
| Platelets count (IQR) | 199.00 [145.00, 264.00] | 199.00 [145.00, 262.00] | 0.867 |
| WBC(IQR) | 10.30 [7.50, 14.20] | 10.30 [7.50, 14.40] | 0.915 |
| RDW(IQR) | 14.30 [13.20, 15.80] | 14.30 [13.30, 15.90] | 0.234 |
| PT(IQR) | 13.50 [12.00, 16.10] | 13.60 [12.10, 16.20] | 0.254 |
| APTT(IQR) | 30.10 [26.60, 36.00] | 30.10 [26.60, 35.95] | 0.836 |
| **First Day Status** |  |  |  |
| Antibiotic Application(%) | 6104 (58.4) | 2684 (59.9) | 0.104 |
| Renal Replacement Therapy(%) | 380 ( 3.6) | 173 ( 3.9) | 0.542 |
| Invasive Line(%) | 6416 (61.4) | 2787 (62.2) | 0.396 |
| Vasoactive(%) | 3439 (32.9) | 1468 (32.7) | 0.852 |
| Ventilation(%) | 3910 (37.4) | 1707 (38.1) | 0.463 |
| **Pre-comorbidities** |  |  |  |
| Myocardial Infarct(%) | 1930 (18.5) | 827 (18.4) | 0.988 |
| Congestive Heart Failure(%) | 3208 (30.7) | 1435 (32.0) | 0.12 |
| Peripheral Vascular Disease(%) | 1361 (13.0) | 632 (14.1) | 0.083 |
| Cerebrovascular Disease(%) | 1991 (19.1) | 890 (19.9) | 0.269 |
| Dementia(%) | 358 ( 3.4) | 176 ( 3.9) | 0.145 |
| Chronic Pulmonary Disease(%) | 2800 (26.8) | 1178 (26.3) | 0.519 |
| Rheumatic Disease(%) | 371 ( 3.6) | 148 ( 3.3) | 0.474 |
| Peptic Ulcer Disease(%) | 268 ( 2.6) | 114 ( 2.5) | 0.982 |
| Paraplegia(%) | 670 ( 6.4) | 289 ( 6.4) | 0.968 |
| Renal Disease(%) | 2275 (21.8) | 1017 (22.7) | 0.228 |
| Malignant Cancer(%) | 1315 (12.6) | 538 (12.0) | 0.332 |
| Metastatic Solid(%) Tumor(%) | 615 ( 5.9) | 279 ( 6.2) | 0.449 |
| Aids(%) | 57 ( 0.5) | 31 ( 0.7) | 0.342 |
| Liver Disease(%) | 1007 ( 9.6) | 412 ( 9.2) | 0.408 |
| Diabetes(%) | 3165 (30.3) | 1344 (30.0) | 0.714 |
| **Outcomes** |  |  |  |
| Sepsis(%) | 4914 (47.0) | 2177 (48.6) | 0.091 |
| ICU LOS (days) (IQR) | 3.21 [2.45, 4.81] | 3.19 [2.49, 4.88] | 0.659 |
| In-hospital mortality(%) | 909 ( 8.7) | 401 ( 8.9) | 0.652 |

Note: Abbreviations: SAPS II: simplified acute physiology score II.CCI: Charlson Comorbidity Index. GCS: Glasgow Coma Scale. SOFA: sepsis-related organ failure assessment. SBP: Systolic arterial pressure. DBP: diastolic blood pressure. MAP: mean arterial pressure. WBC: White blood cells count. RDW: Red blood distribution width.PT: prothrombin time. APTT: activated partial thromboplastin time.ICU LOS: Length of stay in the intensive care unit. Liver disease included mild to severe liver disease.

**Supplementary Table 4. Univariate Analysis the Characteristic of BSI and No-BCgroup in the Training cohort.**

| **Characteristic** | BSI group | No-BC group | z.x^2^ | P-value |
| --- | --- | --- | --- | --- |
| **Numbers** | 999 | 9448 |  |  |
| **Age(years)** | 64.00[52.00, 73.00] | 67.00 [56.00, 78.00] | 70.85 | <0.001 |
| **Sex(male,%)** | 582 ( 58.3) | 5199 (55.0) | 3.69 | 0.055 |
| **Weight(kg)** | 79.90 [67.00, 96.05] | 78.00 [65.40, 93.00] | 73.92 | 0.015 |
| **Scoring system/index** |  |  |  |  |
| SAPS II(IQR) | 42.00 [33.00, 52.00] | 34.00 [26.00, 42.00] | 78.29 | <0.001 |
| CCI(IQR) | 6.00 [4.00, 8.00] | 6.00 [4.00, 8.00] | 74.68 | <0.001 |
| GCS(IQR) | 12.00 [7.00, 14.00] | 14.00 [13.00, 15.00] | 67.81 | <0.001 |
| SOFA(IQR) | 8.00 [5.00, 11.00] | 4.00 [2.00, 6.00] | 81.25 | <0.001 |
| **Laboratory indicators** | |  |  |  |
| Heart Rate(bpm) (IQR) | 96.00 [81.00, 111.00] | 83.00 [73.00, 96.00] | 78.06 | <0.001 |
| MAP(IQR) | 78.00 [67.00, 90.00] | 83.00 [73.00, 96.00] | 70.48 | <0.001 |
| Respiratory Rate(IQR) | 20.00 [16.00, 25.00] | 17.00 [15.00, 21.00] | 77.38 | <0.001 |
| Temperature(IQR) | 36.83 [36.44, 37.28] | 36.67 [36.39, 36.94] | 76.33 | <0.001 |
| Spo2(IQR) | 98.00 [95.00, 100.00] | 98.00 [96.00, 100.00] | 71.4 | <0.001 |
| Glucose(IQR) | 132.0[104.5, 172.5] | 130.0 [106.0, 166.0] | 73.42 | 0.479 |
| Anion gap(IQR) | 16.00 [13.00, 19.00] | 14.00 [12.00, 17.00] | 76.53 | <0.001 |
| Bicarbonate(IQR) | 21.00 [18.00, 25.00] | 23.00 [21.00, 26.00] | 69.62 | <0.001 |
| Bun(IQR) | 26.00 [16.00, 42.00] | 19.00 [13.00, 28.00] | 76.68 | <0.001 |
| Chloride (IQR) | 102.0 [98.0, 107.0] | 104.0 [100.0, 108.0] | 71.37 | <0.001 |
| Creatinine(IQR) | 1.20 [0.80, 2.10] | 0.90 [0.70, 1.30] | 76.81 | <0.001 |
| Sodium(IQR) | 137.0 [134.0, 140.5] | 139.0 [136.0, 141.0] | 70.69 | <0.001 |
| Potassium(IQR) | 4.20 [3.70, 4.70] | 4.20 [3.80, 4.60] | 72.6 | 0.037 |
| Hematocrit(IQR) | 31.40 [26.50, 36.50] | 33.80 [28.60, 38.90] | 70.74 | <0.001 |
| Hemoglobin(IQR) | 10.10 [8.70, 12.00] | 11.10 [9.30, 12.90] | 70.57 | <0.001 |
| Platelets count (IQR) | 185.0 [107.5, 258.0] | 200.0 [147.0, 264.0] | 71.41 | <0.001 |
| WBC(IQR) | 12.30 [8.10, 18.10] | 10.20 [7.50, 13.90] | 75.81 | <0.001 |
| RDW(IQR) | 15.60 [14.00, 17.40] | 14.20 [13.20, 15.60] | 78.14 | <0.001 |
| PT(IQR) | 15.30 [13.30, 19.70] | 13.40 [12.00, 15.80] | 78.15 | <0.001 |
| APTT(IQR) | 32.90 [28.30, 41.65] | 29.90 [26.50, 35.40] | 76.27 | <0.001 |
| **First Day Status** |  |  |  |  |
| Antibiotic Application(%) | 770 ( 77.1) | 5334 (56.5) | 157.31 | <0.001 |
| RenalReplacement Therapy(%) | 127 ( 12.7) | 253 ( 2.7) | 256.71 | <0.001 |
| Invasive Line(%) | 772 ( 77.3) | 5644 (59.7) | 116.55 | <0.001 |
| Vasoactive(%) | 403 ( 40.3) | 3036 (32.1) | 27.18 | <0.001 |
| Ventilation(%) | 493 ( 49.3) | 3417 (36.2) | 66.48 | <0.001 |
| **Pre-comorbidities** |  |  |  |  |
| Myocardial Infarct(%) | 152 (15.2) | 1778 (18.8) | 7.55 | 0.006 |
| Congestive Heart Failure(%) | 307 (30.7) | 2901 (30.7) | 0.00 | 1.00 |
| Peripheral Vascular Disease(%) | 131 (13.1) | 1230 (13.0) | 0.00 | 0.972 |
| Cerebrovascular Disease(%) | 159 (15.9) | 1832 (19.4) | 6.85 | 0.009 |
| Dementia(%) | 34 (3.4) | 324 ( 3.4) | 0.00 | 1.00 |
| Chronic Pulmonary Disease(%) | 253 (25.3) | 2547 (27.0) | 1.15 | 0.284 |
| Rheumatic Disease(%) | 37 (3.7) | 334 ( 3.5) | 0.03 | 0.854 |
| Peptic Ulcer Disease(%) | 55 (5.5) | 213 ( 2.3) | 36.91 | <0.001 |
| Paraplegia(%) | 76 (7.6) | 594 ( 6.3) | 2.41 | 0.121 |
| Renal Disease(%) | 276 (27.6) | 1999 (21.2) | 21.82 | <0.001 |
| Malignant Cancer(%) | 179 (17.9) | 1136 (12.0) | 27.99 | <0.001 |
| Metastatic Solid Tumor(%) | 67 (6.7) | 548 (5.8) | 1.18 | 0.277 |
| Aids(%) | 11 (1.1) | 46 (0.5) | 5.20 | 0.023 |
| Liver Disease(%) | 261 (26.1) | 746 (7.9) | 342.64 | <0.001 |
| Diabetes(%) | 350 ( 35.0) | 2815 (29.8) | 11.50 | 0.001 |

Note: Abbreviations: SAPS II: simplified acute physiology score II.CCI: Charlson Comorbidity Index. GCS: Glasgow Coma Scale. SOFA: sepsis-related organ failure assessment. SBP: Systolic arterial pressure. DBP: diastolic blood pressure. MAP: mean arterial pressure. WBC: White blood cells count. RDW: Red blood distribution width.PT: prothrombin time. APTT: activated partial thromboplastin time. Liver Disease includes mild to severe liver disease.

**Supplementary Figure 2. LASSO coefficient profiles of the 40 texture features.**


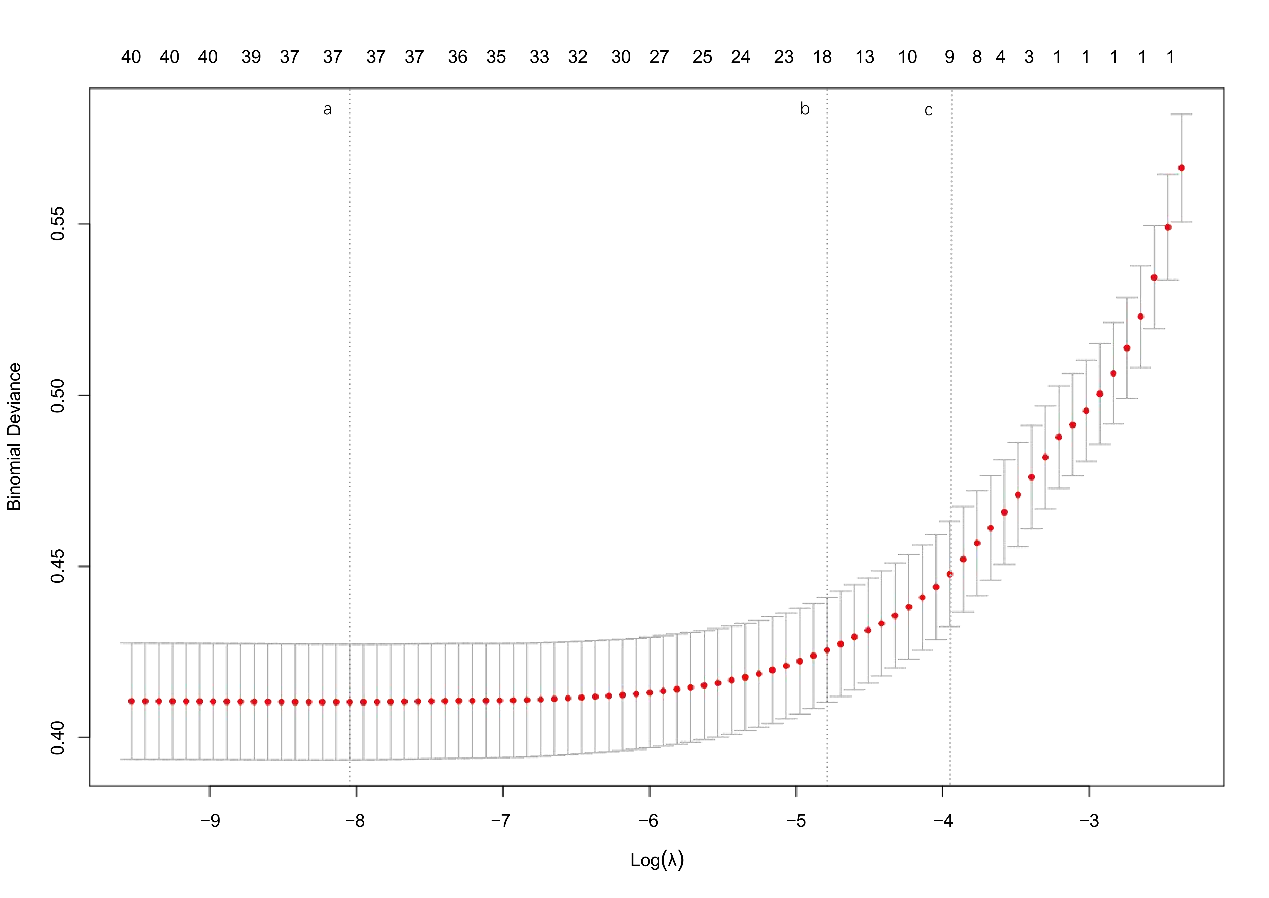


Note: A coefficient profile plot was generated against the log (l) sequence. The vertical line was drawn at the value selected by 10-fold cross-validation, and the dashed vertical lines were drawn at the optimal values using the minimum criteria(a),1 standard error of the minimum criteria (the 1-SE criteria)(b) and the contraction coefficient Lambda=0.025 criteria(c).

**Supplementary Figure 3. ROC curves of the BFH validation dataset and eICU validation dataset with patients admission ICU ≥48h.**


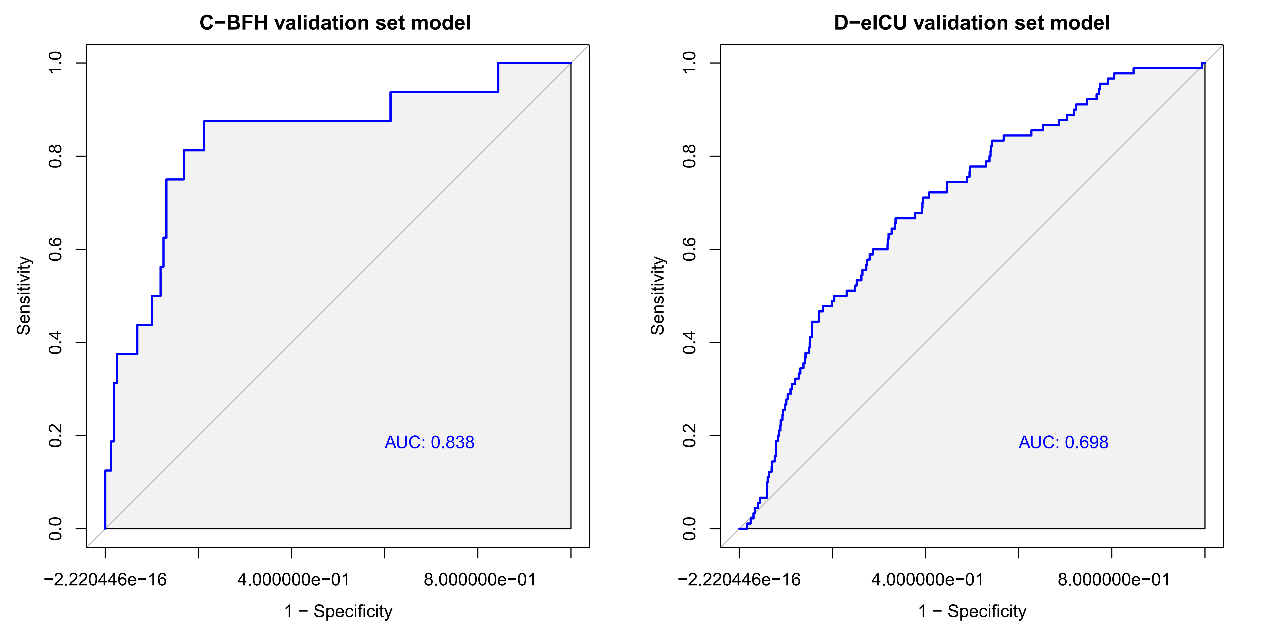

Supplement: Supplementary file 2 [file DataSheet_2.docx]
